# The gut-lung axis in severe pneumonia-related lung injury: mechanisms and therapeutic strategies

**DOI:** 10.3389/fimmu.2025.1700534

**Published:** 2026-01-19

**Authors:** Qiongling Sun, Jun Gao, Xuan Zhao, Tianyi Wang, Wensen Pan, Jing Yu

**Affiliations:** Second Department of Respiratory and Critical Care Medicine, The Second Hospital of Hebei Medical University, Shijiazhuang, China

**Keywords:** fecal microbiota transplantation, gut microbiota dysbiosis, gut-lung axis, microbiota-immune-metabolic interaction, severe pneumonia-related lung injury, short-chain fatty acids

## Abstract

Severe pneumonia-related lung injury is a life-threatening condition associated with high morbidity and mortality. Recent advances in research on the gut-lung axis have provided novel insights into its pathophysiology and revealed potential therapeutic targets. Within the conceptual framework of the microorganism-immunity-metabolism network, modulation of the gut microbiota has emerged as a promising strategy for intervention. Therapeutic approaches such as probiotics, prebiotics, synbiotics, and fecal microbiota transplantation aim to enhance microbial diversity and alter metabolite profiles, thereby optimizing immune responses and attenuating systemic and pulmonary inflammation. This review explores the mechanistic underpinnings of the gut-lung axis in the context of severe pneumonia-related lung injury, with a particular focus on microbiota-targeted interventions. Our goal is to provide a theoretical foundation for the clinical application of gut microbiota modulation in the prevention and treatment of severe pneumonia-related lung injury.

## Graphical Abstract

**Figure:**
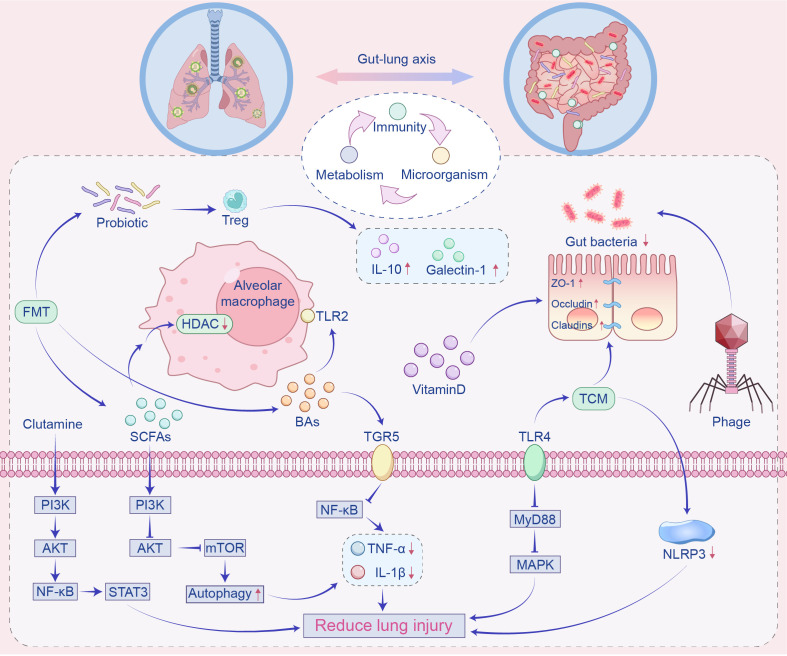
The gut-lung axis in severe pneumonia-related lung injury: mechanisms and therapeutic strategies. Severe pneumonia-related lung injury has high morbidity and mortality. Research on the gut-lung axis provides new therapeutic targets. Modulating the gut microbiota with probiotics, phage therapy, short-chain fatty acids, fecal microbiota transplantation, and traditional Chinese medicine may optimize immune responses and reduce inflammation. IL-10 (Interleukin-10), TLR2 (Toll-like Receptor 2), TGR5 (TGR5 Bile Acid Receptor), TNF-α (Tumor Necrosis Factor-alpha), IL-1β (Interleukin-1 beta), TCM (Traditional Chinese Medicine), and ZO-1 (Zonula Occludens-1 Protein).

## 1 Introduction

Severe pneumonia-related lung injury is a common, life-threatening condition in clinical practice, associated with high incidence and mortality rates (1). Global epidemiological evidence indicates that its incidence is rising, particularly among elderly and immunocompromised populations. Although advances in antibiotic therapy and respiratory support have improved survival, substantial challenges remain in clinical management, underscoring the need for novel therapeutic approaches.

Previous studies have demonstrated that the gut microbiota-immune-metabolic network plays a pivotal role in regulating pulmonary inflammation. Metabolites produced by the gut microbiota, particularly short-chain fatty acids (SCFAs), attenuate pulmonary inflammation by activating G protein-coupled receptors (2). In addition, gut microbial composition and its metabolites modulate the differentiation and function of type 17 helper T cell (Th17) and regulatory T cell (Treg), thereby affecting pulmonary immune homeostasis (3). Patients with severe pneumonia often exhibit gut dysbiosis, which perturbs intestinal homeostasis and exacerbates pulmonary inflammation via the gut-lung axis. The gut-lung axis concept has therefore attracted considerable attention, underscoring the complex interplay between the lungs and the gut microbiota (4). The gut microbiota influences lung health through multiple pathways, including immune modulation and the production of bioactive metabolites (5). However, important gaps remain, including limited understanding of dynamic changes in the gut microbiota during severe pneumonia and the optimal timing of targeted interventions.

This review systematically examines the mechanisms by which the gut-lung axis contributes to severe pneumonia-related lung injury, with a focus on interactions among the gut microbiota, immune pathways, and metabolic signaling, and their collective impact on pulmonary inflammation. It also discusses intervention strategies targeting the gut-lung axis, including modulation of the gut microbiota, restoration of intestinal barrier integrity, metabolic reprogramming, and combination therapies for the management of severe pneumonia-related lung injury.

## 2 Biological basis of the gut-lung axis

### 2.1 Anatomical and physiological connections

The lungs and gastrointestinal tract share notable similarities in embryonic development and histological architecture (6). During gastrulation, the totipotent epiblast differentiates into ectoderm, endoderm, and mesoderm, thereby establishing the foundation for organogenesis (7). The lungs, stomach, and intestines, all derived from endoderm, share structural homology (8, 9). Furthermore, the mucosal surfaces of the respiratory and gastrointestinal tracts are innervated by vagal afferent sensory neurons (10). Burns et al. (11) demonstrated that the intrinsic innervation of the lungs and intestines originates from vagal neural crest cells, confirming their shared embryological lineage.

Beyond their common embryonic origins, the lungs and intestines are functionally connected through complex immune interactions. Immune cells can migrate bidirectionally along the gut-lung axis, trafficking between mucosal sites (12). Zhao et al. (13) used single-cell RNA sequencing to profile innate lymphoid cells in murine lungs and intestines. They found that lung-derived group 2 innate lymphoid cells (ILC2s) express the chemokine receptor CCR2, whereas intestinal ILC2s express CCR4, reflecting tissue-specific imprinting. After interleukin-33 stimulation, CCR2^+^ lung ILC2s migrate to the intestine via the gut-lung axis and transition into CCR4^+^ intestinal ILC2s. Transitional ILC2s co-expressing CCR2 and CCR4 were detected in the intestine, confirming the migratory conversion. Lineage-tracing experiments further verified the ability of lung-derived ILC2s to colonize the intestine. Conversely, Huang et al. (14) showed that intestinal ILC2s can migrate to the lungs through the mesenteric lymphatics, precipitating lung inflammation. This reciprocal immune-cell trafficking exemplifies the physiological link between the lungs and intestines and constitutes a key mechanism of cross-organ immune regulation along the gut-lung axis.

### 2.2 Core regulatory elements

#### 2.2.1 Microbiome

Gut microbiota dysbiosis is typically characterized by reduced microbial diversity and depletion of beneficial taxa, which increases susceptibility to respiratory infections (15). Prophylactic administration of Faecalibacterium duncaniae in mice attenuates influenza-induced weight loss, lowers pulmonary viral load, ameliorates lung and intestinal pathology, and decreases secondary systemic bacterial infections. This protection is closely associated with restoration of SCFAs levels to their pre-infection baseline (16). Following a reassessment based on genomic taxonomy, Faecalibacterium duncaniae has been confirmed as a novel species within the original Faecalibacterium prausnitzii species complex. The widely studied strain A2-165, which was historically classified as F. prausnitzii, has now been reclassified as F. duncaniae.

#### 2.2.2 Immune axis

The complex bidirectional crosstalk between the gut microbiota and immune cells is essential for maintaining immune homeostasis and modulating systemic responses. Treg attenuate pulmonary inflammation, whereas Th17 aggravate lung injury (17). However, the role of Th17 cells is not entirely negative. As a key member of the intestinal fungal microbiota, Candida species can induce the activation of Th17 cells, which, through systemic immune regulation, enhance pulmonary immunity against both bacterial and fungal infections. Th17 cells secrete interleukin-17 and interleukin-22, which recruit neutrophils and activate pulmonary mucosal defenses, thereby aiding in the clearance of pathogenic microorganisms in the lungs (18). Butyrate, a microbial metabolite produced by Faecalibacterium prausnitzii, shifts the Th17/Treg balance toward Treg dominance by inhibiting histone deacetylase (HDAC)-1, thereby exerting strong anti-inflammatory effects (19). In addition, IgA-producing plasma cells are vital for maintaining intestinal microbial equilibrium (20). Gut microbes stimulate B cells to secrete IgA, which enhances pulmonary immune defenses and slows progression of dysbiosis (21).

#### 2.2.3 Metabolic axis

Metabolites such as SCFAs, bile acids (BAs), and tryptophan derivatives constitute a metabolic axis that critically modulates immunity. SCFAs, principally acetate and butyrate, are produced by microbial fermentation of dietary fiber. These molecules regulate immune-cell functions by activating G protein-coupled receptors and inhibiting HDAC, thereby preserving systemic homeostasis (22). Acting as circulating signaling molecules, SCFAs reach the lungs via the portal circulation and modulate pulmonary immune responses. Accordingly, they represent a key metabolic conduit linking the gut microbiota to pulmonary immunity (2).

## 3 Mechanisms of the gut-lung axis in severe pneumonia-related lung injury

### 3.1 Pathogenic effects of gut microbiota dysbiosis

Respiratory viral infections provoke exaggerated immune responses that release pro-inflammatory mediators, recruit immune cells, and ultimately disrupt gut-microbiota homeostasis, thereby inducing intestinal inflammation (15). Concomitant antibiotic therapy can aggravate dysbiosis, intensify virus-induced injury to pulmonary and intestinal tissues, and disturb the Th17/Treg balance, thereby weakening lung immune defenses (23). Zeng et al. (24) reported markedly reduced fecal microbial diversity in antibiotic-treated pneumonia patients relative to healthy controls. Interestingly, Hashimoto et al. (25) showed that antibiotic-induced depletion of gut microbes can reduce pro-inflammatory metabolites, thereby attenuating distal lung inflammation and ameliorating lipopolysaccharide (LPS)-induced acute lung injury. Critical illness further diminishes microbial diversity, reshapes bacterial communities, and favors opportunistic overgrowth, conditions that facilitate gut-to-lung translocation of pathogens (26). Among the taxa implicated, members of the Enterobacteriaceae family are particularly associated with nosocomial lung infections (27). In 31 intensive-care patients, Liu et al. (28) combined metagenomics with flow cytometry and detected high abundances of Enterococcus faecalis in paired bronchoalveolar-lavage and fecal samples. These findings support dysbiosis-driven bacterial translocation and suggest that E. faecalis and other Enterobacteriaceae may aggravate lung injury via the gut-lung axis.

### 3.2 The “gut-derived second hit” hypothesis

**Figure 1:**
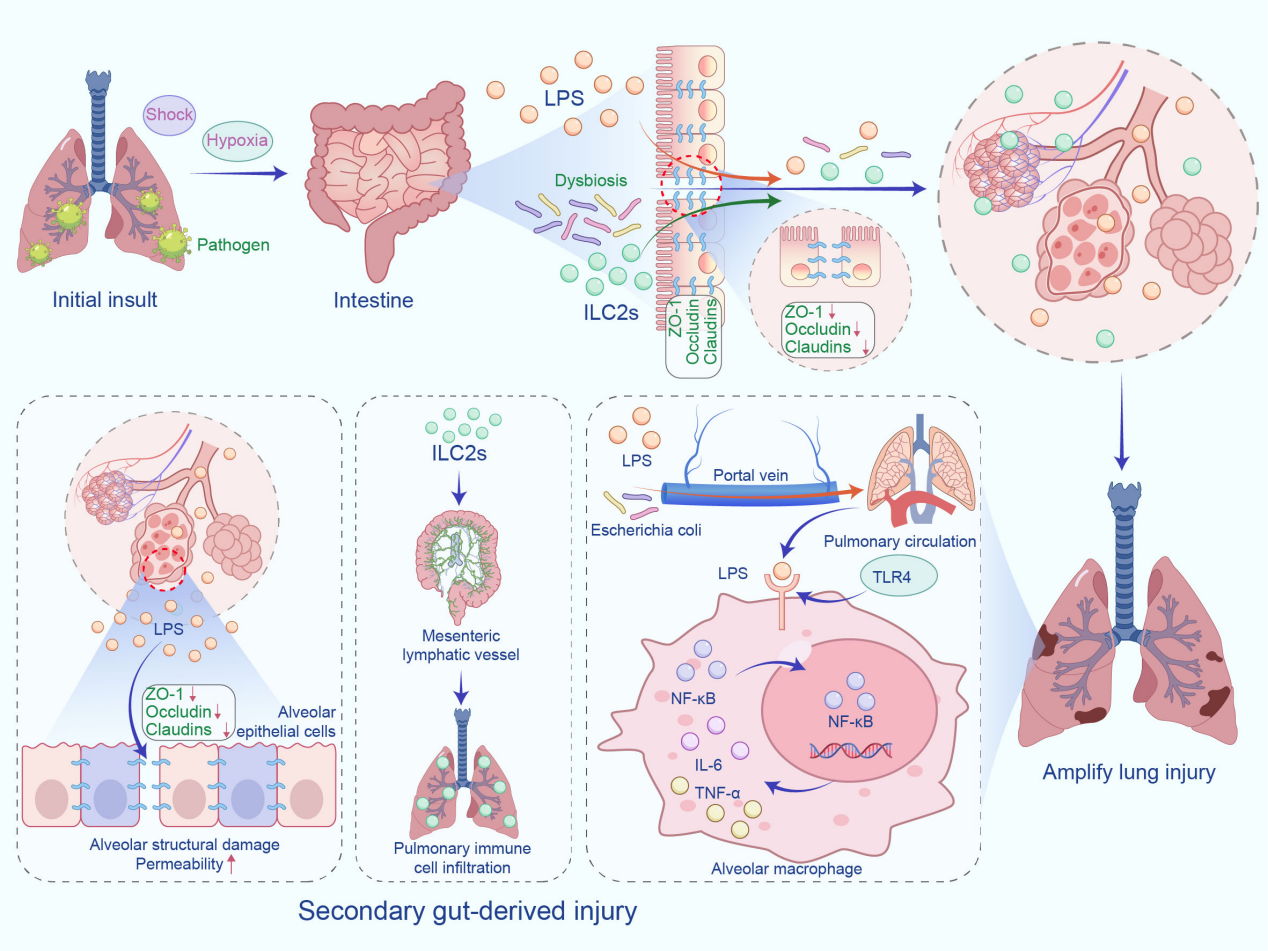
The “gut-derived second hit” hypothesis. In severe pneumonia, lung tissue invasion is the initial insult, with the intestine as a secondary injury source. Stress factors impair the intestinal barrier, causing dysbiosis and LPS translocation, activating TLR4-NF-κB, disrupting lung immune homeostasis, and increasing epithelial permeability, exacerbating damage. IL-6 (Interleukin-6), and TNF-α (Tumor Necrosis Factor-alpha).

In severe pneumonia, primary invasion of lung tissue constitutes the initial insult, whereas the intestine may provide a secondary source of injury. Systemic stressors—including shock, hypoxemia, and broad-spectrum antibiotics—provoke intestinal ischemia-reperfusion injury that compromises both the structural and functional integrity of the mucosal barrier. Barrier failure precipitates dysbiosis, facilitates bacterial translocation, and permits LPS leakage. These gut-derived danger signals reach the systemic circulation through the portal vein and lymphatics, thereby amplifying lung injury (29). Using a cecal ligation-and-puncture model, Bao et al. (30) showed that enteric bacteria such as Escherichia coli traverse the compromised intestinal barrier and colonize the lungs, where they trigger inflammation and tissue damage. However, it is important to note that the prevalence of live bacterial translocation during disease processes still requires substantial evidence to support it and remains difficult to confirm. LPS, a potent Toll-like receptor-4 (TLR4) ligand derived from Gram-negative bacteria, activates the TLR4-nuclear factor-κB (NF-κB) pathway (31). Subsequent signaling prompts alveolar macrophages to secrete pro-inflammatory mediators, drives TLR4 desensitization, and disturbs pulmonary immune equilibrium (32, 33). LPS also directly impairs alveolar epithelial cells by down-regulating tight-junction proteins—including zonula occludens-1 and occludin—thereby decreasing cell viability and increasing permeability (34). Claudins—a family of tight-junction transmembrane proteins—are indispensable for alveolar homeostasis. Consistent with this, LaFemina et al. (35) found that Claudin-18-deficient mice display barrier disruption, impaired alveolarization, and parenchymal destruction, underscoring the pivotal role of this protein in lung integrity. The migration of gut-derived immune cells and the spread of inflammatory mediators can also affect lung physiology (36). A study by Eladham et al. (37) found that disruption of intestinal integrity in an inflammatory bowel disease animal model promotes microbial translocation and aberrant immune cell homing, leading to pulmonary inflammation. The proposed mechanism is illustrated in **Figure 1**.

### 3.3 Bidirectional regulation of microbial metabolites

#### 3.3.1 Protective metabolites

SCFAs produced by gut microbes can traverse the circulation and modulate immune microenvironments in distant organs, including the lungs. Such systemic signaling enhances antiviral immunity and strengthens host defenses against respiratory viral infections (38). SCFAs can directly affect type II alveolar epithelial cells by activating the cell surface receptors free fatty acid receptor 2 and free fatty acid receptor 3. This activation inhibits LPS-induced NF-κB signaling pathway activation, reduces the release of pro-inflammatory factors such as interleukin-1β, and helps maintain the immune homeostasis of type II alveolar epithelial cells (39). Moreover, a study by Jiang et al. (40) showed that butyrate, via HDAC inhibition, increases histone acetylation at the promoter of nuclear factor interleukin-3 regulated, thereby up-regulating nuclear factor interleukin-3 regulated expression. Elevated nuclear factor interleukin-3 regulated suppresses cytokine production in ILC2s and markedly alleviates ILC2s-mediated pulmonary inflammation. Zhu et al. (3) likewise demonstrated that butyrate lowers HDAC activity in lung tissue, promotes Treg differentiation, and mitigates inflammation triggered by polycyclic aromatic hydrocarbons. By acting as an HDAC inhibitor, butyrate exerts broad epigenetic effects that influence host physiology and health (41). Rectal butyrate administration also attenuates pulmonary fibrosis in mice, partly by activating the HDAC-peroxisome proliferator-activated receptor-γ axis and inducing hepatocyte growth factor expression in the colon (42).

#### 3.3.2 Harmful metabolites

Not all microbial metabolites are beneficial to the lung. Hydrogen sulfide (H_2_S), as a microbial metabolite, exerts effects on the lungs that are closely related to its concentration. Elevated concentrations of H_2_S damage pulmonary epithelial cells, promote inflammatory-cell infiltration, increase cytokine release, and culminate in lung injury (43). It is important to note that low concentrations of H_2_S have a protective effect on the lungs. Ge et al. (44) demonstrated in a rat model of ventilator-associated lung injury that inhalation of low-concentration H_2_S significantly improved respiratory function, alleviated pathological damage such as alveolar septal thickening and inflammatory cell infiltration, and effectively reduced the severity of lung injury. However, some studies suggest that long-term, continuous exposure to H_2_S, even at lower concentrations, may still lead to lung damage. Li et al. (45) showed that long-term inhalation of low concentrations of H_2_S concentrations activate immune pathways, skew the balance between pro-inflammatory and anti-inflammatory mediators, and disturb tissue homeostasis. Tumor necrosis factor and chemokine signaling cascades appear central to this process, further exacerbating lung inflammation. Immune hyperactivation thus initiates an inflammatory cascade that disrupts the air-blood barrier, leading to lung injury and, potentially, pulmonary fibrosis.

## 4 Intervention strategies targeting the gut-lung axis

### 4.1 Microbiome remodeling

#### 4.1.1 Probiotics, prebiotics, and synbiotics

Probiotic strains belonging to the genera Lactobacillus and Bifidobacterium confer antiviral benefits primarily through immune modulation. These bacteria reduce pro-inflammatory mediators, enhance antiviral immunity, and broadly temper inflammatory processes. In addition, probiotics can directly inactivate certain viruses and curb the growth of opportunistic bacteria (46). Accordingly, probiotic supplementation represents a promising adjunctive therapy for critically ill patients (47). For example, Zhu et al. (3) showed that Lactobacillus murinus mitigates pulmonary inflammation and restores the Th17/Treg balance by regulating host tryptophan metabolism and SCFAs production. Similarly, Shen et al. (48) reported that Lactobacillus supplementation lessens LPS-induced lung injury, attenuates inflammatory signaling, and reduces macrophage and neutrophil infiltration. Chen et al. (49) further confirmed that Lactobacillus johnsonii supplementation alleviates respiratory viral infection in mice while exerting anti-inflammatory effects. Albarracin et al. (50) showed that immunomodulatory lactic-acid bacteria augment respiratory interferon responses, rebalance pro-inflammatory and anti-inflammatory cytokines, limit bacterial replication, and ameliorate lung damage. Vogel et al. (51) found that T-cell priming with Bifidobacterium promotes Treg differentiation, increasing secretion of interleukin-10 and galectin-1. This cascade suppresses Staphylococcus-specific Th17 activation through a cell-intrinsic mechanism and depends on cytotoxic T lymphocyte-associated protein 4 signaling. It is important to note that probiotic therapy for severe diseases has notable limitations. In many intensive care unit patients, the gut microbiota exhibits enhanced antibiotic resistance, resulting in significant individual variability in microbiota composition. This variability significantly hampers the clinical effectiveness of probiotics (52).

Mannan-oligosaccharide acts as a prebiotic, selectively promoting the growth of beneficial genera such as Bifidobacterium and Lactobacillus while inhibiting pathogens like Escherichia coli and Salmonella. Such modulation helps preserve intestinal microbial homeostasis. Recent studies indicate that mannan-oligosaccharide reduces bacterial adhesion and enhances antibiotic efficacy, providing a novel antibacterial strategy (53). Human-milk oligosaccharides function as soluble decoy receptors, blocking viral, bacterial, and protozoan attachment to epithelial glycans and thereby preventing respiratory infection (54). Both human-milk oligosaccharides and galacto-oligosaccharides enrich Bifidobacterium populations in the gut microbiota (55). Zhu et al. (56) demonstrated that galacto-oligosaccharides exerts potent antibacterial activity against both macrolide-resistant and macrolide-sensitive Mycoplasma pneumoniae. Consequently, galacto-oligosaccharides both combats infection and supports microbial homeostasis.

Synbiotic supplementation mitigates LPS-induced declines in lung function, reduces pulmonary neutrophilia, and improves the neutrophil-to-lymphocyte ratio (57). Sjödin et al. (58) further reported that synbiotics decrease Klebsiella abundance, elevate Bifidobacterium levels, and enhance microbial metabolites linked to immune signaling along the gut-lung axis. These findings support the concept that synbiotics promote integrated respiratory-gut homeostasis. Compared to severe pneumonia-associated lung injury, the therapeutic effects of probiotics, prebiotics, and synbiotics in chronic lung diseases, such as cystic fibrosis, are particularly closely related to the duration of treatment. A study by Bruzzese et al. (59) on children with cystic fibrosis showed that six months of probiotic supplementation significantly reduced the risk of acute pulmonary exacerbations and improved lung function. However, research by Rahmani et al. (60) indicated that 12 weeks of short-term probiotic use did not show significant effects on lung function or acute exacerbations in children with cystic fibrosis. Although short-term treatment did not demonstrate notable improvements, long-term use may offer potential benefits in reducing acute exacerbations and improving lung function. Future studies should further explore the optimal treatment duration and dosage to provide more effective management strategies for patients with chronic lung diseases.

Although the mechanisms of probiotic, prebiotic, and synbiotic therapies for severe pneumonia-associated lung injury are promising in basic research, evidence from randomized controlled trials in humans remains inconsistent. In critically ill patients requiring mechanical ventilation, probiotics did not result in significant differences in the occurrence and progression of ventilator-associated pneumonia when compared to placebo (61). However, preventive synbiotic therapy has been shown to significantly reduce the incidence of ventilator-associated pneumonia in sepsis patients by modulating the gut microbiota and intestinal environment (62). Therefore, future research should focus on the effects of specific strains, optimal timing of treatment, and personalized treatment strategies to better optimize therapeutic approaches for critically ill patients.

#### 4.1.2 Fecal microbiota transplantation

FMT has emerged as a promising therapy for a range of severe conditions (63). The procedure seeks to re-establish microbial equilibrium and thereby preserve gut integrity (64). FMT shows particular promise for severe pneumonia complicated by extensively drug-resistant bacterial infections. Zhuang et al. (65) described a 95-year-old patient with severe COVID-19 pneumonia and extensively drug-resistant Klebsiella pneumoniae infection whose pathogen cleared and clinical status improved after FMT. Their report demonstrated that FMT reshaped the gut microbiota, increased beneficial taxa, suppressed K. pneumoniae, and modulated immunity via SCFAs. However, due to its classification as level V evidence, the generalizability of these findings is limited. Therefore, further multi-center prospective cohort studies are needed to validate the efficacy and safety of this treatment in severe pneumonia-associated lung injury. Tang et al. (66) showed that FMT enriches beneficial metabolites—including secondary BAs—reduces systemic inflammation, restores alveolar-epithelial integrity, and attenuates lung injury. Wen et al. (67) further observed that FMT rebalances the Treg/Th17, suppresses systemic inflammation, and alleviates Pseudomonas aeruginosa-induced pulmonary damage. It is important to note that long-term antibiotic use in patients with severe pneumonia can lead to gut microbiota dysbiosis. Therefore, when using FMT for the treatment of severe pneumonia, the impact of gut microbiota heterogeneity on treatment outcomes should be considered, and personalized treatment approaches may be necessary.

#### 4.1.3 Phage therapy

Phage therapy has demonstrated significant therapeutic potential for severe pneumonia-associated lung injury. Ashworth et al. (68) found that phage cocktail therapy significantly reduced the lung bacterial load in a mouse model of severe pneumonia caused by pan-drug-resistant Pseudomonas aeruginosa. Early administration even completely cleared lung infections in some mice and alleviated bacterial-mediated lung damage. Furthermore, in the context of pneumonia-induced lung injury, phage therapy not only effectively clears lung infections and prevents bacteremia but also mitigates excessive inflammatory responses and can rapidly correct hematological abnormalities caused by the infection. Its safety and efficacy are comparable to those of traditional antibiotics (69). However, most existing studies are animal experiments or small-scale clinical case reports, lacking large-scale randomized controlled trials to validate long-term efficacy and safety. Therefore, clinical evidence still needs to be further developed.

### 4.2 Intestinal barrier repair

Maintenance of intestinal-barrier integrity is critical for systemic homeostasis. Bioactive compounds and their signaling pathways not only repair the intestinal barrier but also provide novel targets for precise intervention in severe pneumonia-related lung injury.

Glutamine, a conditionally essential amino acid, participates in numerous physiological processes. It is a precursor for glutathione and neurotransmitters and directly supports intestinal-barrier structure. By modulating tight-junction protein expression, glutamine precisely regulates paracellular permeability (29). Multiple studies confirm that glutamine, as an immunonutrient, preserves intestinal-epithelial integrity. For example, Liu et al. (70) showed that alanyl-glutamine ameliorates dextran-sulfate-sodium-induced colitis in mice and concomitant lung injury by modulating the gut microbiota and PI3K-Akt/NF-κB/STAT3 signaling. Similarly, Wang et al. (71) demonstrated that glutamine metabolism is indispensable for alveolar-epithelial regeneration during lung injury, and clinical supplementation markedly alleviates severe respiratory symptoms.

Vitamin D promotes intestinal-barrier repair by up-regulating tight-junction proteins, thereby strengthening epithelial integrity (72). Lee et al. (73) showed that vitamin D supplementation suppresses tumor necrosis factor-α production, restores tight junctions, and reduces epithelial permeability, establishing an anti-inflammatory repair pathway.

TLR4 plays a crucial role in initiating the innate immune response by recognizing LPS (74). Over-activation of TLR4 provokes excessive inflammation, disrupts intestinal-barrier integrity, and worsens pneumonia pathophysiology. Accordingly, precise TLR4 modulation is a key strategy for mitigating gut-lung inflammatory damage. Targeting TLR4/NF-κB signaling effectively tempers immune activation and alleviates pneumonia-related inflammation (75). Lambertucci et al. (76) demonstrated that benznidazole mitigates inflammation by down-regulating TLR4 and activating Nrf2. Small-molecule TLR4 inhibitors have also shown promising efficacy in animal models. Fan et al. (77) reported that pinitol ameliorates LPS-induced pneumonia by inhibiting TLR4 and NF-κB/IκBα signaling.

### 4.3 Metabolite intervention

#### 4.3.1 Exogenous SCFAs supplementation

SCFAs produced by gut microbes modulate both acute and chronic respiratory diseases by activating G protein-coupled receptors and inhibiting HDAC (2). Notably, the SCFAs butyrate acts as an intrinsic HDAC inhibitor and potently regulates inflammatory-gene expression (78). In a murine model of vancomycin-induced dysbiosis, Kabil et al. (79) showed that oral SCFAs supplementation—especially butyrate—reversed the aggravated pneumonia phenotype. Park et al. (80) reported that butyrate both indirectly and directly exerts anti-fibrotic effects—by modulating macrophage differentiation and inhibiting HDAC3—thereby ameliorating pulmonary fibrosis. Sapra et al. (81) observed that SCFAs attenuate pulmonary inflammation by targeting neutrophils, promoting phagolysosome release, and inducing neutrophil-extracellular-trap formation. He et al. (82) showed that sodium propionate ameliorates LPS-induced acute respiratory distress syndrome in rats by suppressing the PI3K/AKT/mTOR pathway, enhancing autophagy, reducing inflammatory-mediator release, and protecting alveolar epithelial cells. Hildebrand et al. (83) demonstrated that SCFAs treatment dampens inflammatory-signal amplification in aged mice with acute lung injury. SCFAs also contribute importantly to antiviral immunity: Antunes et al. (84) showed that they enhance antiviral defenses, lower viral load, and suppress pro-inflammatory responses during rhinovirus infection.

#### 4.3.2 Metabolic intervention with BAs and related compounds

BAs function as pleiotropic signaling molecules that activate receptors such as the farnesoid-X receptor (FXR) and the G protein-coupled bile acid receptor, thereby influencing numerous physiological and pathological processes. Agonists of these BAs receptors markedly modulate pulmonary inflammation. Among them, the nuclear receptor FXR has received significant attention. FXR activation protects intestinal-epithelial integrity and also orchestrates host metabolism in concert with the gut microbiota (85). Gadaleta et al. (86) showed that activation of the FXR-FGF19 axis promotes BAs turnover, reduces intestinal inflammation, and reshapes microbiota composition. Bear bile powder (BBP) mainly contains tauroursodeoxycholic acid and sodium taurocholate. Tauroursodeoxycholic acid, the principal bioactive constituent of BBP, exhibits well-documented lung-protective effects. Cheng et al. (87) demonstrated that BBP lowers myeloperoxidase activity and inflammatory-cytokine levels and down-regulates CD14, thereby inhibiting NF-κB activation. Histology further showed that BBP attenuates neutrophil infiltration and prevents cell desquamation, necrosis, and alveolar collapse in lung tissue. Tong et al. (88) found that BBP reshapes gut microbiota and increases SCFAs production; moreover, auroursodeoxycholic acid alone alleviated bleomycin-induced alveolar-septal destruction and inflammatory infiltration. Beyond auroursodeoxycholic acid, additional BAs also modulate pulmonary inflammation. Gong et al. (89) showed that taurochenodeoxycholic acid markedly reduces alveolar oedema and neutrophil infiltration following Staphylococcus aureus infection and significantly lowers high-mobility group box 1 expression in lung tissue. Mechanistically, taurochenodeoxycholic acid decreases inflammatory-mediator levels in lung and serum, suppresses pro-inflammatory cytokine release, and inhibits mitogen-activated protein kinase, NF-κB, and Toll-like receptor 2 signaling in macrophages. Thus, taurochenodeoxycholic acid -mediated Toll-like receptor 2 regulation may underlie its ability to alleviate lung injury. Mechanistic studies of ursodeoxycholic acid (UDCA) and chenodeoxycholic acid are comparatively advanced. Milivojac et al. (90) showed that UDCA and chenodeoxycholic acid attenuate endotoxin-induced lung injury in rats by regulating aquaporin 1 and aquaporin 5 to maintain water balance, reducing oxidative stress in alveolar epithelium, modulating apoptosis-related proteins (BAX, caspase-3, BCL-2), and inhibiting NF-κB-driven cytokine release. Emerging evidence has further implicated receptor-mediated pathways in the pharmacological actions of UDCA. He et al. (91) reported that UDCA significantly reduces tumor necrosis factor-α and interleukin-1β expression *in vitro*. *In vivo*, UDCA up-regulates FXR while suppressing p38 mitogen-activated protein kinase and NF-κB p65 phosphorylation, thereby dampening inflammation and protecting the lung.

### 4.4 Traditional Chinese medicine treatment

The theory of “the Lung and Large Intestine being interior-exteriorly related” in Traditional Chinese Medicine reveals the close connection between lung and intestinal functions, while the modern “gut-lung axis” mechanism provides scientific support for this traditional understanding. A study by Wang et al. (92) confirmed that the classic Traditional Chinese Medicine formula Yupingfeng San has significant protective effects against LPS-induced acute lung injury. The mechanism of action is through regulating the gut-lung axis for combined lung and intestinal protection: on one hand, Yupingfeng San inhibits the TLR4-MyD88-MAPK pathway and NLRP3 inflammasome activation in lung tissue, reducing alveolar septal thickening, inflammatory infiltration, and pulmonary edema; on the other hand, it upregulates the expression of tight junction proteins, such as Occludin and Claudin-1, in colon tissue, protecting the integrity of the intestinal mucosal barrier, inhibiting the release of intestinal inflammatory factors, and reducing the amplification effect of lung injury caused by gut microbiota translocation. This approach aligns perfectly with the Traditional Chinese Medicine treatment concept of “lung and intestine harmonization.

### 4.5 Combination therapy strategies

Integrated interventions that simultaneously target the microbiome-immune-metabolic axis show promise for managing severe pneumonia-related lung injury. Combining complementary interventions is likely more effective than any single modality. For example, probiotic or prebiotic supplementation modulates microbiota composition, increases diversity, and suppresses pathogen overgrowth (46). In severe pneumonia, FMT can mitigate pulmonary inflammation by restoring eubiotic gut communities (65). Adequate nutritional support reinforces the intestinal barrier and reduces inflammatory-mediator release (70). Microbiota-derived SCFAs further ameliorate lung injury by modulating immune-cell function and damping inflammatory cascades (80).

Therapeutic sequencing aligned with disease stage is recommended in severe pneumonia. During the acute phase, broad-spectrum antibiotics should be initiated promptly and then rationalized once microbiological data are available. Concurrently, enteral nutrition and probiotics support the mucosal barrier and limit bacterial translocation. During recovery, additional probiotic courses or FMT may facilitate convalescence and promote durable microbial resilience.

## 5 Challenges and perspectives

### 5.1 Current limitations

While the combined treatment approach directed at the gut-lung axis holds promise for managing severe pneumonia-related lung injury, there exist notable limitations. Patients with severe pneumonia exhibit marked inter-individual variation in gut microbiome composition, driven by genetic, lifestyle, and environmental factors. Such heterogeneity hinders the design of universally applicable treatment protocols, and identical interventions can therefore yield divergent clinical outcomes. Moreover, the pharmacokinetics of metabolites such as SCFAs complicate translational research. SCFAs are rapidly cleared *in vivo*; for example, butyrate exhibits a plasma half-life of approximately six minutes, making it challenging to sustain therapeutic concentrations (93). Because *in vivo* SCFAs levels fluctuate within a narrow homeostatic range, both excessively high and low concentrations can be harmful. Therefore, personalized strategies tailored to specific patient characteristics are essential (94). Similarly, the application of FMT faces numerous ethical, religious, and safety concerns (95). Therefore, a personalized treatment strategy is essential throughout the entire FMT process. Candidates for FMT should be selected based on clinical, microbial, immunological, and metabolic parameters from blood and stool analyses, with the FMT protocol tailored to the characteristics and disease condition of the recipient to ensure effective colonization of the donor microbiota and therapeutic outcomes. Additionally, continuous monitoring of clinical efficacy, biomarkers, immune parameters, and the composition and function of the microbiota is required post-FMT treatment (96). Critically ill patients often have polymicrobial infections, multiple organ failure, and immunosuppressive states, which are significantly different from animal models that typically involve single-pathogen infections. These factors may influence the clinical efficacy of gut-lung axis-based interventions such as FMT, probiotics, prebiotics, and synbiotics in the treatment of severe pneumonia. Direct translation of findings from animal models to clinical practice remains a substantial challenge.

### 5.2 Future directions

Developing highly representative humanized animal models is a key priority for future research. Improved humanized models would better recapitulate human physiology and pathology. Indeed, a dual-humanized mouse model has already been used to dissect the complex interactions between the gut microbiome and the immune system (97). Dietary recommendations personalized to the microbiome profile of an individual microbiome profile, supplemented with tailored nutraceuticals, could optimize metabolic outcomes. Additionally, engineering microbial strains to over-produce beneficial metabolites—such as SCFAs—may yield novel biotherapeutics capable of correcting dysbiosis and mitigating lung injury in severe pneumonia. Additionally, due to the short half-life of butyrate, various delivery strategies are currently being explored to maintain the necessary drug exposure for therapeutic effect and improve its bioavailability. One such novel delivery platform has been developed, consisting of a methacrylamide backbone, butyrate or benzoyl ester side chains, and a mannose side chain, which allows for the sustained release of butyrate (98). Notably, modifying symbiotic bacteria to produce butyrate *in situ* in the gut is another approach to maintaining endogenous butyrate production (99).

In the future, research should focus on designing differentiated intervention strategies for different stages of severe pneumonia-associated lung injury (hyper-acute, acute, sub-acute, and recovery phases), evaluating their efficacy during each window period. Randomized controlled trials should be conducted, with adjustments to probiotic, prebiotic, or FMT strategies based on microbiota or immunological responses, to determine the optimal timing for each treatment.

## 6 Conclusions

The gut-lung axis is a key determinant of severe pneumonia-related lung injury and therefore constitutes a compelling therapeutic target. Elucidating the complex crosstalk among microbes, immunity, and metabolism provides a framework for rational intervention design. Future studies should delineate mechanistic details and develop precisely tailored treatment protocols to improve outcomes in severe pneumonia.
